# Endoplasmic Reticulum–Mitochondria Calcium Communication and the Regulation of Mitochondrial Metabolism in Cancer: A Novel Potential Target

**DOI:** 10.3389/fonc.2017.00199

**Published:** 2017-09-08

**Authors:** Galdo Bustos, Pablo Cruz, Alenka Lovy, César Cárdenas

**Affiliations:** ^1^Anatomy and Developmental Biology Program, Institute of Biomedical Sciences, University of Chile, Santiago, Chile; ^2^Geroscience Center for Brain Health and Metabolism, Santiago, Chile; ^3^Department of Neuroscience, Center for Neuroscience Research, Tufts University School of Medicine, Boston, MA, United States; ^4^Buck Institute for Research on Aging, Novato, CA, United States; ^5^Department of Chemistry and Biochemistry, University of California, Santa Barbara, Santa Barbara, CA, United States

**Keywords:** inositol triphosphate receptors, mitochondrial Ca^2+^ uniporter, mitochondrial transport, TCA cycle, respiratory chain, AMPK

## Abstract

Cancer is characterized by an uncontrolled cell proliferation rate even under low nutrient availability, which is sustained by a metabolic reprograming now recognized as a hallmark of cancer. Warburg was the first to establish the relationship between cancer and mitochondria; however, he interpreted enhanced aerobic glycolysis as mitochondrial dysfunction. Today it is accepted that many cancer cell types need fully functional mitochondria to maintain their homeostasis. Calcium (Ca^2+^)—a key regulator of several cellular processes—has proven to be essential for mitochondrial metabolism. Inositol 1,4,5-trisphosphate receptor (IP3R)-mediated Ca^2+^ transfer from the endoplasmic reticulum to the mitochondria through the mitochondrial calcium uniporter (MCU) proves to be essential for the maintenance of mitochondrial function and cellular energy balance. Both IP3R and MCU are overexpressed in several cancer cell types, and the inhibition of the Ca^2+^ communication between these two organelles causes proliferation arrest, migration decrease, and cell death through mechanisms that are not fully understood. In this review, we summarize and analyze the current findings in this area, emphasizing the critical role of Ca^2+^ and mitochondrial metabolism in cancer and its potential as a novel therapeutic target.

## Introduction

Mitochondria arose around two billion years ago as the result of a symbiotic interaction between an archaeon ancestor and a α-proteobacterium (1). The modern mitochondria has retained a small circular polycystronic 16 kilobase genome (2) that controls the synthesis of about 67 proteins, including 13 proteins that are core constituents of the electron transport chain (complex I–IV) (3). The terminal complex V of the electron transport chain, the ATP synthase, catalyzes the synthesis of most of the ubiquitous cellular energy currency, ATP (4). In addition, mitochondria play a vital role in metal ion homeostasis (5), programmed cell death (6–8), and the synthesis of building blocks for the generation of amino acids, lipids, and nucleotides (9). In many cancer types, glucose-derived pyruvate is transported away from mitochondria, turned to lactate and excreted (10). Detection of glutamine-derived carbon as lactate has established glutamine as an important energy source in tumor cells (11, 12). Glutamine contributes the bulk of carbon to the tricarboxylic acid (TCA) cycle through a phenomenon known as anaplerosis (13), maintaining a robust amount of citrate and malate for biosynthesis of lipids and nucleotides, respectively, in addition to providing an extra supply of reductive power (NADH) (9, 14). Also, glutamine activation of the TCA cycle helps to maintain the mitochondrial membrane potential (ΔΨm) (15), avoiding opening of the permeability transition pore and the release of pro-apoptotic factors (16). Mitochondria play a pivotal role in maintaining cellular homeostasis, and their function is essential for the viability of cancer cells.

## Mitochondrial Metabolism in Cancer

A hallmark feature of cancer cells is a re-programming of their metabolism even when nutrients are available (17, 18). All major tumor suppressors and oncogenes are now recognized to have fundamental connections with metabolic pathways (10, 19, 20). Warburg suggested that cancer originates from an irreversible injury to mitochondria followed by a compensatory increase of glycolysis (21), but growing evidence indicates that most cancer cells rely on mitochondrial metabolism (20, 22–24). For example, mitochondrial function is essential for the survival of diffuse large B cell tumors (25), primary glioblastoma sphere cultures (26), pancreatic (27), and leukemic cancer (28). Moreover, a subset of cancer cells that exclusively rely on glycolysis for ATP production [with mutations either in components of the succinate dehydrogenase complex or the fumarate hydratase rendering mitochondrial oxidative phosphorylation (OXPHOS) dysfunctional] still use and need the oxidation of alpha-ketoglutarate (α-KG) in the mitochondria to generate the reducing equivalents necessary to sustain the reductive carboxylation pathway and the generation of metabolic intermediates (29). The mitochondrial function in cancer cells is sustained by a high uptake of the non-essential amino acid glutamine (15), which is converted to glutamate by glutaminases and subsequently converted to α-KG by either glutamate dehydrogenases or aminotransferases (30). α-KG then enters the TCA cycle where it is a substrate of the α-KG dehydrogenase (α-KGDH), a highly regulated enzyme that catalyzes the conversion of α-KG, coenzyme A and NAD^+^ to succinyl-CoA, CO_2_ and NADH providing electrons to the respiratory chain (14, 29). Importantly in the context of this review, α-KGDH activity is strongly Ca^2+^ dependent (31). Ca^2+^ also activates directly the isocitrate dehydrogenase (ISDH) and indirectly the pyruvate dehydrogenase (PDH) through regulation of its phosphorylation state (32, 33). In general, increases of Ca^2+^ in the 0.1–10 µM range up-regulates the activities of these enzymes, resulting in higher mitochondrial NADH levels (34, 35). The regulation by Ca^2+^ of these three key mitochondrial dehydrogenases has a strategic task in coordinating cellular workload and generation of ATP (36). Several studies demonstrate that Ca^2+^ signaling plays an important role in cancer progression by promoting proliferation, cell migration, metastasis and vascularization, and conferring apoptosis resistance (37). However, how Ca^2+^ signals may affect mitochondrial metabolism and impact the aforementioned features remains poorly understood. Although Ca^2+^ is the focus of this mini-review, it is not the only ion able to affect mitochondrial metabolism. For example, K^+^ transport modulates the coupling between mitochondrial respiration and ATP synthesis (38). In fact, inhibition of the mitochondrial potassium channel Kv1.3 or the two-pore potassium channel TASK-3 compromises mitochondrial function (39, 40). Mg^2+^ is also important, since the knockdown of Mrs2, a Mg^2+^-selective channel in the inner mitochondrial membrane (IMM), induces the loss of complex I and causes mitochondrial membrane depolarization (41).

## Inositol 1,4,5-Trisphosphate Receptors (IP3Rs) and Cancer

The IP3Rs are a ubiquitous family of Ca^2+^ release channels composed of three isoforms (1, 2, and 3) present primarily in the endoplasmic reticulum (ER) (42) but can also be found in the nuclear envelope where they regulate gene transcription (43, 44). In addition, Ca^2+^ release through the IP3R regulates numerous other cellular functions including secretion, motility and autophagy (42, 45). Agonist-induced IP3R Ca^2+^ signals enhance mitochondrial function (46–48) primarily by stimulating the TCA cycle dehydrogenases (PDH, α-KGDH, and ISDH) (31), as well as respiratory chain components to promote OXPHOS and ATP production (49, 50). The above is possible in part by the spatial proximity that exists between these two organelles, which allows the establishment of a structural and functional coupling known as mitochondria-associated membranes (MAMs). These structures were reported in the early 1950s (51–53) and have been described as signaling platforms involved in many cellular processes including metabolism control, migration, differentiation, proliferation and cell death (54). Interestingly, many oncosupressors and oncogene proteins are located in the MAMs and interact with the IP3R (such as AKT, PML, PTEN, and mTORC2) modifying calcium release patterns and cellular fate, which has been thoroughly discussed elsewhere (55, 56).

In cancer, the expression of IP3Rs, in particular the IP3R-3 isoform, is up-regulated in glioblastoma (57), gastric (58), small and non-small lung (59), and colorectal cancer (60). Importantly, the over-expression of IP3R-3 in gastric cancer was found in cell lines established from cells that invade the peritonea, while the ones made from primary tumor cells show normal levels of IP3R expression (58). Along these lines, in colorectal cancer IP3R-3 was found in the advancing margins of the tumors, correlating with depth of invasion, lymph node metastasis and liver metastasis (60). In glioblastoma, the inhibition of IP3R with caffeine, a non-specific inhibitor of the IP3R, decreased migration in various *in vitro* assays and increased mean survival in a mouse xenograft model of glioblastoma (57). In addition, siRNA silencing of IP3R-3 in the colon cancer cell line CACO-2, or non-specific pharmacological inhibition of IP3R by 2APB in gastric cancer cells, induces apoptosis, while over-expression of the receptors protects the cells from apoptosis induced by staurosporine (60). Regarding breast cancer, both estradiol- and ATP-induced proliferations in the MCF7 cell line are mediated by the IP3R-3 (61, 62). Moreover, the breast cancer metastasis suppressor 1, a protein able to suppress formation of secondary tumor masses without blocking growth of neoplastic cells at orthotopic or subcutaneous sites, reduces phosphoinositide signaling, including IP3 in the MDA-MB-435 cell line (63). Clearly, IP3R plays a role in cancer progression and metastasis; however, the mechanism of its involvement has not been elucidated. A summary of the expression of IP3R in cancer and the effect of manipulating its activity is found in Table 1.

**Table 1:** Effects of inositol 1,4,5-trisphosphate receptor (IP3R) or MCUC component modulation on cancer cells.

Protein	Expression levels or activity modulation	Outcome reported	Tumor types or cell lines	Reference
IP3R	Up-regulation	Possible involvement in dissemination	Gastric cancer, colorectal cancer	(58, 60)
		Alters endoplasmic reticulum-calcium homeostasis	Lung carcinoma cell lines	(59)
	Caffeine inhibition	Reduces of invasion and extends survival	Glioblastoma	(57)
	Knockdown	Cell death	CACO-2	(60)
			MCF7, T47D, PC3, DU145	(65)
		Decreases estradiol-induced proliferation	MCF7	(61)
	XeB inhibition	Cell death	MCF7, T47D, PC3, DU145	(65)

MCU	Down-regulation	Provide cancer cell survival upon apoptotic challenges	Colon cancer	(87)
	Knockdown	Reduces migration and metastasis	MDA-MB-231	(91)
		Potentiates caspase-independent cell death	MDA-MB-231	(88)
		No effects on cell survival	MDA-MB-231	(89)
		Cell death	Transformed primary skin fibroblast	(65)
	Knockdown or Ruthenium red inhibition	Reduces migration (involves store-operated Ca^2+^ entry)	MDA-MB-231	(90)
	Enhances activity	Cell death	Hela, EA.hy926	(94)

MICU1	Knockdown	Reduces migration and sensitizes to apoptotic stimulus	HeLa	(95)
		Sensitizes to apoptotic stimulus	Melanoma, head and neck squamous cell carcinoma	(96, 97)
		Inhibits tumor growth, migration and invasion	Ovarian cancer	(98)

MCUR1	Knockout	Increases resistance to cell death	HeLa	(100)

**Figure 1:**
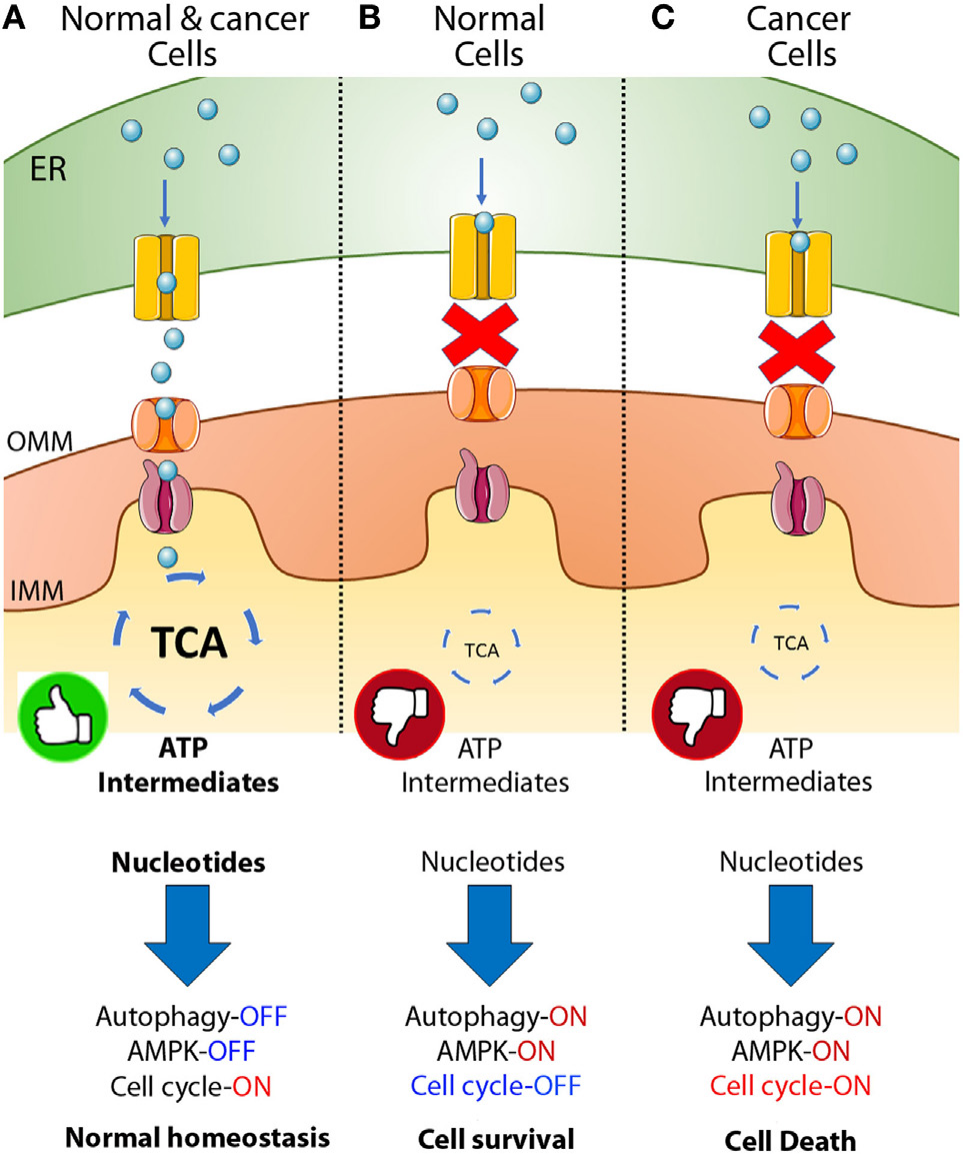
Inositol 1,4,5-trisphosphate receptor (IP3R)-mediated calcium transfer to mitochondria in normal and cancer cells. **(A)** In normal and cancer cells Ca^2+^ released from the endoplasmic reticulum (ER) through the IP3R enters the mitochondrial matrix through the mitochondrial calcium uniporter (MCU) and activates key dehydrogenases of the tricarboxylic acid (TCA) cycle, maintaining a robust amount of ATP and metabolic intermediates or building blocks for the generation of fatty acids, amino acids and nucleotides allowing the cells to enter the cell cycle, proliferate, and keep normal homeostasis. **(B)** In normal cells, the inhibition of the Ca^2+^ transfer to mitochondria generates a decrease in TCA cycle activity with the concomitant reduction in ATP and metabolic intermediates, inducing AMPK, autophagy and the complete shutdown of the cell cycle caused in part by a reduction in the availability of nucleotides. **(C)** In cancer, a similar phenomenon is observed after inhibition of Ca^2+^ transfer to mitochondria; decrease in TCA cycle activity, ATP, and metabolic intermediates, activation of AMPK and autophagy, and reduction in the amount of nucleotides available. However, cancer cells continue entering the cell cycle despite the metabolically unfavorable environment causing cell death (65, 66).

Constitutive IP3R-mediated Ca^2+^ transfer to mitochondria is essential to maintain cell bioenergetics in normal cells, and its absence induces a bioenergetic stress that causes diminished oxidative respiration (OXPHOS) and leads to an adaptive response characterized by AMPK and autophagy activation (Figure 1A) (64). We find that breast and prostate cancer derivate cells, as well as transformed primary fibroblasts, similar to normal cells need constitutive transfer of IP3R released Ca^2+^ to the mitochondria to maintain the optimal activity of PDH and, therefore, sufficient amounts of NADH to support the TCA cycle (Figure 1A) (unpublished data). Genetic or pharmacological inhibition of either IP3R or MCU elicits comparable effects to those observed in normal cells, including diminished OXPHOS, AMPK activation, and induction of a pro-survival autophagy (Figures 1B,C); however, whereas normal cells were able to survive these challenges, much of the cancer cell population (60–70%) could not (Figure 1C). The cancer cell death was caused by a lack of mitochondrial function because mitochondrial substrates, which bypassed the inhibition of the TCA cycle and enabled mitochondria to work, were able to rescue the cells (65, 66). Moreover, we determined that a decrease in nucleotides, whose synthesis occurs partially in the mitochondria, added to the inability of cancer cells to arrest the cell cycle upon metabolic stress and was most likely responsible for the mitotic catastrophe that leads to cell death in cancer cells, as supplementation with nucleotides promoted survival.

## MCU and Cancer

In the early 1960s, it was observed that energized mitochondria were capable of taking up and accumulating Ca^2+^ in the matrix (67–69) and further studies found that mitochondrial Ca^2+^ uptake could be pharmacologically inhibited by ruthenium red and Ru360 (70–72). Nevertheless, this mitochondrial feature was ignored for many years and, only in the 1990s, did mitochondrial Ca^2+^ uptake begin to be considered an important mechanism for Ca^2+^ homeostasis (73–75). The capacity to take up and accumulate Ca^2+^ in the mitochondrial matrix is substantial, reaching concentrations of 100 µM (5). Nevertheless, most of the Ca^2+^ that enters the mitochondrial matrix is quickly chelated due to the action of phosphate; therefore, free [Ca^2+^] within the matrix is much lower compared to total [Ca^2+^], which can reach up to 1 M, as occurs in neurons. According to the chemiosmotic theory of Mitchell, the entrance of Ca^2+^ into the mitochondrial matrix is driven by the negative potential inside the membrane (76). However, exactly how Ca^2+^ enters the mitochondrial matrix was a critical question unsolved for more than 50 years.

Ca^2+^ crosses the outer mitochondrial membrane through the voltage-dependent anion-selective channels (VDACs), a family of channels by which different ions and molecules enter into the intermembrane space (77). VDACs play a significant role in cancer by supporting glycolytic metabolism and preventing apoptosis, which has been comprehensively reviewed recently elsewhere (78). Ca^2+^ then enters the mitochondrial matrix through the so-called uniporter, whose molecular identity was unknown until recently (79, 80). The discovery of the regulatory subunit mitochondrial Ca^2+^ uptake 1 (MICU1) was the first step in this field, discovered in 2010 by Mootha’s group, who used siRNA screening of over 13 candidates and identified an IMM protein of 54 kDa necessary for mitochondrial Ca^2+^ uptake (81). A year later, Mootha’s and Rizzuto’s group in parallel identified a 40 kDa protein as the pore-forming element of the uniporter, the mitochondrial calcium uniporter (MCU) (79, 80). These findings radically opened further studies, and today, we know that the mitochondrial uniporter is a multi-protein complex (MCUC) formed by MCU, MCUb, EMRE (core components of the pore) (82–84), MICU1 and 2 (81, 85), MCUR1 (86), and SLC25A23 (86) (associate regulators).

The role of the MCUC in cancer is just beginning to be explored and already with contradicting results (see Table 1) For example, the reduced expression of MCU caused by over-expression of the microRNA miR-25 in colon cancer favors cancer cell survival by preventing mitochondrial Ca^2+^ overload upon several apoptotic challenges (87). In contrast, several groups, using various different algorithms, find that the expression of MCU correlates with a poor prognosis, invasive behavior, and metastasis in breast cancer (88–91). Zou’s group shows that knockdown of MCU decreases migration through a mechanism that implies a reduction of the store-operated Ca^2+^ entry (90); however, the molecular details of this mechanism remain unclear. More recently, Tosatto et al. elegantly show that MCU silencing decreases migration and metastasis through a mechanism that includes an increase in the NAD(P)H/NADH ratio, changing the antioxidant capacity of the cell and decreasing the steady-state levels of mitochondrial reactive oxygen species (ROS). This in turn affects the stabilization of Hypoxia-inducible factor-1α (HIF-1α), with the concomitant decrease in its target genes such as the hexokinase II, the glucose-6-phosphate isomerase and the lysyl oxidase, which are critical for migration (91). The change in the NAD(P)H/NADH cannot be explained solely as the result of a decrease in the TCA cycle flux suggesting the presence of other Ca^2+^-sensitive mechanisms in the mitochondria that need to be explored.

The knockdown of MCU in the highly aggressive breast cancer cell line MDA-MB-231 does not affect proliferation or cell viability (88–91), unless the cells are challenged with a caspase-independent cell death inducer such as ionomycin, which shows that MCU knockdown cells are significantly more sensitive than MCU expressing cells (88). Interestingly, in transformed primary skin fibroblasts, the knockdown of MCU induces a massive cell death (64), perhaps reflecting cell type-specific differences regarding the role of MCU or the use of the mitochondria, since the MDA-MB-231 cells are highly glycolytic while the skin primary fibroblasts are more oxidative. Along these lines, primary endothelial cells derived from tissue-specific MCU or MCUR1 null mice also show a reduction in migration but, contrary to work in breast cancer, also show reduction in proliferation that correlates with the decrease in mitochondrial Ca^2+^ uptake and impaired mitochondrial bioenergetics (92). Interestingly, in endothelial cell lines, the oxidation of MCU Cys-97 enhances MCU channel activity increasing mitochondrial Ca^2+^ uptake and ROS generation. The persistent elevation of mitochondrial Ca^2+^ and ROS generation harms mitochondrial bioenergetic function, reducing migration and sensitizing cells to death (93). Also, a decrease in sarco/ER calcium ATPase (SERCA) activity in HeLa and EA.hy926 cancer cell lines caused by a drop in mitochondrial ATP levels (using resveratrol, piceatannol, or oligomycin) enhanced mitochondrial Ca^2+^ uptake causing Ca^2+^ overload and apoptosis, specifically in cancer cells (94).

The knockdown of MICU1, which is contrary to the MCU or MCUR1 knockdown, causes a constitutive entry of Ca^2+^ to the mitochondria, also reduced migration, and sensitized cells to apoptotic stimuli (95). In agreement, in melanoma, one of the most aggressive and lethal cancers, the knockdown of the ribosomal protein S3, reduced the expression of MICU1 allowing a mitochondrial Ca^2+^ overload that triggered apoptosis (96). Similarly, the inhibition of the enhancer of zeste homolog 2, a component of the Policomb repressive complex 2, involved in the regulation of homeotic (Hox) gene expression in head and neck squamous cell carcinoma downregulated MICU1 and caused cell death (97). Along these lines, MICU1 expression in ovarian cancer decreased PDH activity and hence the TCA cycle, shifting the cells toward glycolysis, providing chemoresistance but leading to a poor overall survival. Silencing MICU1 increases OXPHOS and inhibits tumor growth, migration, and invasion (98). Physiologically, MICU1 is a gatekeeper for MCU-mediated Ca^2+^ uptake, establishing a threshold that prevents Ca^2+^ uptake when [Ca^2+^] is low (<3 µM), similar to that observed during rest or during weak agonist stimulation. At higher [Ca^2+^] MICU1 allows the entry of Ca^2+^, essential to maintain cellular bioenergetics and viability (95). In fact, a recent publication of Graier group has demonstrated that cancer cells use the uncoupling proteins 2 and 3 (UCP2/3) to re-establish MICU1 function (and in turn mitochondrial Ca^2+^ uptake), which is desensitized by methylation mediated by PRMT1 (99), an arginine methyl transferase highly expressed in cancer (100). The presence of this compensatory mechanism confirms the importance of preserved mitochondrial function in cancer. Finally, MCUR1 knockdown inhibits cell death induced by mitochondrial Ca^2+^ overload and is involved in establishing the mitochondrial permeability transition (101) and, therefore, it will be interesting to determine its role in cancer. Clearly, fine-tuning of mitochondrial calcium is fundamental for mitochondrial performance and ultimately cellular fate, and it will be exciting to further decipher the role of the MCUC in cancer.

## Ryanodine Receptor (RyRs) and SERCAs in Cancer

The RyRs and the SERCA are the major Ca^2+^ players in the ER together with the IP3R (102). RyRs are a family of Ca^2+^-release channels composed of three isoforms (1, 2, and 3) that have rarely been associated with cancer, despite the fact that they regulate proliferation of melanocytes and T cells (103, 104) and migration in astrocytes (105). Implications in apoptosis regulation in prostate cancer cell line LNCaP (106) and a direct correlation with tumor grades on breast cancer (107) are the strongest findings so far. It is possible that the limited participation of RyR in MAMs and Ca^2+^ communication with mitochondria prevents them from participating more actively in cancer.

In contrast, SERCA, consisting of three isoforms, such as SERCA1–2, which is expressed differentially in several tissues, and SERCA3, which is ubiquitously expressed (108), has been broadly associated with different types of cancer. Alteration of SERCA expression has been reported in oral squamous cancer (109), choroid plexus (110), thyroid (111), lung (59, 112), colon (113, 114), acute promyelocytic leukemia (108), cervical (115), and breast cancers (116). In colon cancer, SERCA3 expression decreases progressively during the tumorigenesis process becoming virtually null in poorly differentiated tumors (114). Similarly, ductal carcinomas show absence of SERCA3 expression compared to their normal counterparts (116) as well as acute promyelocytic leukemia cells (108) and Burkitt’s lymphoma cells (117), the details of which have been discussed thoroughly elsewhere (37, 108, 118).

## Conclusion

Warburg was the first to propose a link between mitochondria and cancer by suggesting that the origin of cancer cells was an irreversible lesion in mitochondria that raises the glycolytic rate as a compensatory mechanism. Today, increasing evidence has demystified Warburg’s theory, demonstrating that mitochondria are key actors for cancer biology. To support their uncontrolled proliferation rate, cancer cells need a constant supply of building blocks to feed biomass production. This is achieved by a metabolic re-programming that favors certain metabolic pathways and in this context; mitochondrial metabolism has proven to be essential. Accumulating evidence points to the Ca^2+^ communication between the ER and mitochondria as key for mitochondrial function and, therefore, for cancer progression. Unfortunately, the limited access of inhibitors either for IP3R or for MCU has prevented the development of pharmacokinetics and pharmacodynamics experiments *in vivo*, hindering the understanding of the real potential of this pathway as a therapeutic option, as has been achieved for other ion channels and the SERCA pump (118) Thus, the design of new drugs targeting the functional Ca^2+^ coupling between ER and mitochondria is fundamental to further understand the role of this pathway in phenomena such as angiogenesis, metastasis, chemoresistance, and cancer relapse.

## Author Contributions

GB, PC, and CC designed and outlined the structure and contents of the review. GB, PC, AL, and CC contributed to the literature review, discussion, and writing of the manuscript. All authors contributed equally to the draft revisions and final approval of the version to be published.

## Conflict of Interest Statement

The authors declare that the research was conducted in the absence of any commercial or financial relationships that could be construed as a potential conflict of interest.
